# MicroRNAs in B-cells: from normal differentiation to treatment of malignancies

**DOI:** 10.18632/oncotarget.3057

**Published:** 2014-12-10

**Authors:** Sara Correia Marques, Maria Bach Laursen, Julie Støve Bødker, Malene Krag Kjeldsen, Steffen Falgreen, Alexander Schmitz, Martin Bøgsted, Hans Erik Johnsen, Karen Dybkaer

**Affiliations:** ^1^ Department of Haematology, Aalborg University Hospital, Aalborg, Denmark; ^2^ Department of Clinical Medicine, Aarhus University, Denmark; ^3^ Department of Clinical Medicine, Aalborg University, Denmark; ^4^ Clinical Cancer Research Center, Aalborg University Hospital, Denmark

**Keywords:** MicroRNA, B-cell, Differentiation, B-cell malignancies, Drug response

## Abstract

MicroRNAs (miRNAs) are small non-coding RNAs that play important post-transcriptional regulatory roles in a wide range of biological processes. They are fundamental to the normal development of cells, and evidence suggests that the deregulation of specific miRNAs is involved in malignant transformation due to their function as oncogenes or tumor suppressors. We know that miRNAs are involved in the development of normal B-cells and that different B-cell subsets express specific miRNA profiles according to their degree of differentiation. B-cell-derived malignancies contain transcription signatures reminiscent of their cell of origin. Therefore, we believe that normal and malignant B-cells share features of regulatory networks controlling differentiation and the ability to respond to treatment. The involvement of miRNAs in these processes makes them good biomarker candidates. B-cell malignancies are highly prevalent, and the poor overall survival of patients with these malignancies demands an improvement in stratification according to prognosis and therapy response, wherein we believe miRNAs may be of great importance. We have critically reviewed the literature, and here we sum up the findings of miRNA studies in hematological cancers, from the development and progression of the disease to the response to treatment, with a particular emphasis on B-cell malignancies.

## INTRODUCTION

MicroRNAs (miRNAs) are central players in the regulation of cellular processes from proliferation and differentiation to metabolism and apoptosis [1]. They are fundamental to the normal development of cells and evidence implies that miRNA deregulation is involved in the pathogenesis of human diseases. Translational research has identified candidate miRNA biomarkers for use in diagnosis and treatment guidance in a broad range of diseases, including hematological cancers [2, 3].

Most non-Hodgkin lymphomas (NHL) are considered to be derived from germinal center (GC) cells based on histological features, immunohistochemical characteristics, and immunoglobulin (Ig) gene rearrangements. Implementation of global gene expression profiling (GEP) has allowed for a more comprehensive phenotype-based assignment to a cell of origin (COO) classification, reminiscent of the normal B-cell differentiation program. In diffuse large B-cell lymphoma (DLBCL), GEP-based COO classification recognizes three main subclasses: GC B-cell-like (GCB) DLBCL, which represents transformed GC centroblasts (CB) [4, 5]; activated B-cell-like (ABC) DLBCL, which resembles post-GC plasmablasts [6]; and primary mediastinal large B-cell lymphoma (PMBCL), thought to originate from a thymic post-GC B-cell [7-9]. In agreement with different messenger RNA (mRNA) profiles, distinct miRNA signatures are associated with molecular subclasses of DLBCL [10-13]. In support of the GEP-based subclassifications, there are observations of recurrent genetic abnormalities with subclass-specific distributions and activation of distinct signaling pathways [14]. Thus, each naturally occurring differentiation subset of B-cells has intrinsic gene and miRNA phenotypic profiles granting them specific abilities to further differentiate, respond to stimuli, and survive when exposed to drugs. These features can be partly maintained when a malignant condition initiates in a cellular differentiation-specific compartment. The malignancy then either develops by expansion of that same end-stage compartment or differentiates into a more mature one. We believe the classification of NHL can be further refined using GEP and miRNA expression profiles determined in normal B-cell differentiation subsets isolated from healthy human secondary lymphoid tissue [15, 16]. This idea has prompted us to look into the translational research of miRNAs and their use in the characterization of normal and malignant B-cell subsets.

This review will present examples of the most recent miRNA translational studies. We will focus on the impact of miRNAs on the development and progression of hematological malignancies, especially from the B-cell lineage, and on their potential as biomarkers of prognosis and response to chemotherapy.

### BIOGENESIS AND ACTIVITY OF miRNAs

#### miRNA biogenesis

**Figure 1:**
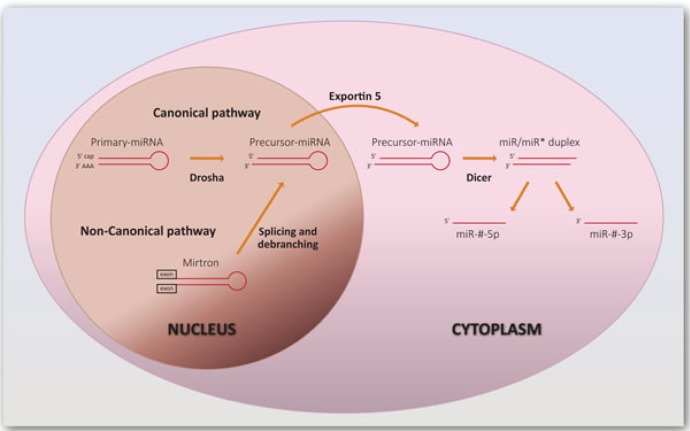
Biogenesis of miRNAs The synthesis of miRNAs via the canonical pathway starts with transcription of miRNA genes by RNA polymerase II, which results in primary-miRNAs. These are processed by Drosha and DiGeorge syndrome critical region gene 8 (DGCR8), generating precursor-miRNAs which are transported to the cytoplasm by Exportin 5 and cleaved by Dicer and TAR RNA-binding protein (TRBP). The resulting duplex is separated, generating mature miRNAs. MiRNAs can also arise from splicing through the non-canonical pathway. The designation of miRNAs includes the term “miR” preceding a number attributed sequentially. Similar sequences can have the same number but a different suffix (number or letter). Letters defining the species are added as prefixes, such as hsa for *Homo sapiens*. Additionally, they can be designated miR-#-3p or miR-#-5p depending on which arm of the precursor structure the leading strand is located [168].

The synthesis of mature and fully competent miRNAs is designated miRNA biogenesis, a process that begins in the nucleus and is completed in the cytoplasm (Figure 1). In the canonical biogenesis pathway, intergenic miRNA genes are transcribed by RNA polymerase II into 5′-capped and 3′-polyadenylated molecules, the primary-miRNAs [17]. These are processed by a protein complex comprising Drosha and DiGeorge syndrome critical region gene 8 (DGCR8), generating intermediates of approximately 70 nucleotides (nts) called precursor-miRNAs, which contain an approximately 2 nts long 3′ overhang [18]. Precursor-miRNAs are transported to the cytoplasm by Exportin 5 [19] and cleaved by a complex that includes the RNAse enzyme Dicer and TAR RNA-binding protein (TRBP), resulting in a miRNA duplex [20-23]. The duplex is separated originating mature miRNAs, which are 20 to 25 nts long single-stranded RNAs. One of the strands, the passenger strand, is usually degraded and the other, called the guide strand, is loaded into the RNA-induced silencing complex (RISC) with Argonaute (Ago) proteins, enabling the targeting of mRNAs through complementary base pairing [24].

Another less common process of synthesizing miRNAs arises from splicing. This non-canonical pathway leads to the formation of mirtrons, which are miRNAs encoded in introns with hairpin-forming potential. They are not cleaved by Drosha, but are spliced out directly by the spliceosome complex, and they enter the biogenesis at the precursor stage [25, 26].

#### miRNA activity

A single miRNA can target hundreds of mRNAs [27], and every mRNA can be regulated by several miRNAs [28]. This confers onto these small RNAs a broad scope of action, affecting a multitude of cellular transcripts and pathways.

The miRNA guide strand can be either the 3′ or the 5′ of the precursor stem-loop structure, meaning that both sequences have the potential to become mature miRNAs. Strand selection for loading into the RISC can be either protein-mediated or based on thermodynamic stability [29]. Both strands are capable of functional activity, and the passenger strand, also called miRNA* or complementary strand, has also been documented to be involved in human disease [30-33].

Mature miRNAs bind to their target mRNA through partially or fully complementary base pairing primarily in the 3′ untranslated region (UTR) of the mRNA [34]. Most frequently, this induces inhibition of translation or mRNA degradation, which can be mediated by P-bodies [35]. However, miRNAs can also bind to their targets and act in other ways, as exemplified by miR-223, which binds to the promoter region of *NFI-A* and inhibits its transcription, reducing both mRNA and protein levels [36], and by the *de novo* DNA methylation of the promoter region of *hoxd4* by miR-10a, resulting in transcriptional downregulation [37]. Regulatory functions through the targeting of the open reading frame of mRNAs mediating repression have also been reported [38-41].

MiRNAs can also activate translation and help stabilize viral mRNA, such as in the function of miR-122 towards the hepatitis C virus [42-45]. Additionally, they can be directly regulated by other RNAs, as suggested by the competing endogenous RNA (ceRNA) hypothesis from Paolo Pandolfi's group [46]. In summary, the better-known mechanism of action of miRNAs is the degradation or inhibition of translation of their target mRNA(s). However, miRNAs can also upregulate mRNA translation, can be modulated by mRNAs and other non-coding RNAs, and can be responsible not only for post-transcriptional but also transcriptional regulation.

### B-CELL DIFFERENTIATION

B-cells undergo a stepwise differentiation process initiating from hematopoetic stem cells located in the bone marrow, where they differentiate into precursor B-cells [47]. This maturation process is characterized by a rearrangement of the V (variable), D (diversity), and J (joining) gene segments of the Ig genes. When the B-cell antigen receptor (BCR), comprising two identical heavy-chain and two light-chain Ig polypeptides, has been tested for auto-reactivity, the naïve B-cells leave the bone marrow and migrate via the blood to the secondary lymphoid tissues. Here, GCs are formed upon an encounter between the BCR and a foreign antigen [48-50]. In the GC a dark and a light zone can be distinguished. The dark zone consists mainly of proliferating CB undergoing somatic hypermutation whereas centrocytes (CC) are located in the light zone. The differentiation of CB and CC includes several rounds of migration between the dark and the light zones. A re-encounter between the B-cell and the antigen in a T-cell and follicular dendritic cell-dependent manner within the light zone ensures increased affinity between the Ig and the antigen. Following optimal antibody selection, a shift in the effector function by class switch DNA recombination (CSR) takes place in the CC in the light zone. The B-cells then leave the GC as memory B-cells or plasmablasts [49, 51, 52].

#### MiRNAs in B-cell differentiation

MiRNAs are fundamental to the development of blood cells, capable of regulating almost every stage of hematopoiesis [53] with lineage and differentiation-specific expression [54]. They are important determinants of B-cell maturation [55], and different stages of normal B-cell differentiation are characterized by different miRNA expression profiles [56-58].

When the expression of Dicer or members of the Ago family are removed, the synthesis of mature miRNAs in mouse models is impaired and B-cell differentiation is affected, highlighting the importance of miRNAs in the formation of B-cells [59]. When Dicer is ablated, early transition from pro-B to pre-B-cells [55], formation of GC B-cells [60], and terminal B-cell differentiation [61] are blocked. Thus, it is clear that antigen-dependent activation is not the sole driver of the formation of effector B-cells; their maturation is also highly dependent on the regulatory role of miRNAs.

Selectively targeting and manipulating the expression of miRNAs allowed the determination of their function at specific steps of B-cell differentiation. One of the first miRNAs identified in this manner was miR-181 (present name miR-181a-5p). Ectopic overexpression of this miRNA in hematopoietic stem-progenitor cells caused an increased fraction of B-cells in both tissue culture differentiation assays and adult mice [62]. The fact that miR-181a-5p is highly expressed in early human CD34+ hematopoietic stem-progenitor cells [63] and is downregulated in pre-BII [57] is indicative of an important function in early B-cell development. Additionally, it is predicted to inhibit differentiation of all hematopoietic lineages in an integrative bioinformatics analysis of miRNA and mRNA expression in human stem-progenitor cells from bone marrow and peripheral blood [63]. These findings are in accordance with studies in human immature precursor B-cell subsets, where miR-181a-5p was found to inversely correlate with the differentiation inhibitor ID2 mRNA, supporting a regulatory role in early differentiation of B-cells [57].

Like miR-181a-5p, manipulation of miR-150 in ectopic expression studies has provided insight into its role in normal B-cell differentiation. When miR-150 is overexpressed in murine hematopoietic stem-progenitor cells, the transition from pro-B to pre-B-cell is blocked and mature B-cells are reduced in numbers. MiR-150-knockout mice displayed a fourfold expansion of mature B1 cells, which are innate immune cells, combined with the enhanced apoptosis of pro-B-cells [64, 65]. Therefore, in normal B-cell differentiation, expression of miR-150 is high in the mature B-cells and relatively low in the immature ones, and is especially decreased at the pro-B to pre-B-cell transition [53, 64, 66]. The primary target of miR-150 is c-Myb, a transcription factor controlling multiple steps of B-cell development. Manipulation of the expression of miR-150 fits well with affected c-Myb function because the transition from pro-B to pre-B-cells requires c-Myb, which is consequently indirectly necessary for the generation of mature B-cells [65].

MiR-155 is highly expressed in GC B-cells and relatively low levels are observed in hematopoetic stem cells and mature B-cells [63, 67, 68]. It plays an important role in the control of the GC reaction, as documented by an increased number of GC B-cells in transgenic mice overexpressing this miRNA compared to knockout mice [69]. MiR-155-knockout mice had a reduced memory B-cell response when challenged by antigen immunization [69, 70] and B-cells lacking miR-155 presented reduced levels of high-affinity IgG1 antibodies [71]. Involvement of miR-155 in the regulation of activation-induced cytidine deaminase expression during affinity maturation and CSR in the GC, together with miR-181b, further support the role of miR-155 in B-cell differentiation [72, 73].

The cluster miR-17-92 has also been shown to be expressed in GC cells, and miR-125b, miR-9, and members of the miR-30 family are overexpressed in the GC compared to plasma cells, showing evidence of their involvement in the differentiation of B-cells [11, 58].

**Figure 2:**
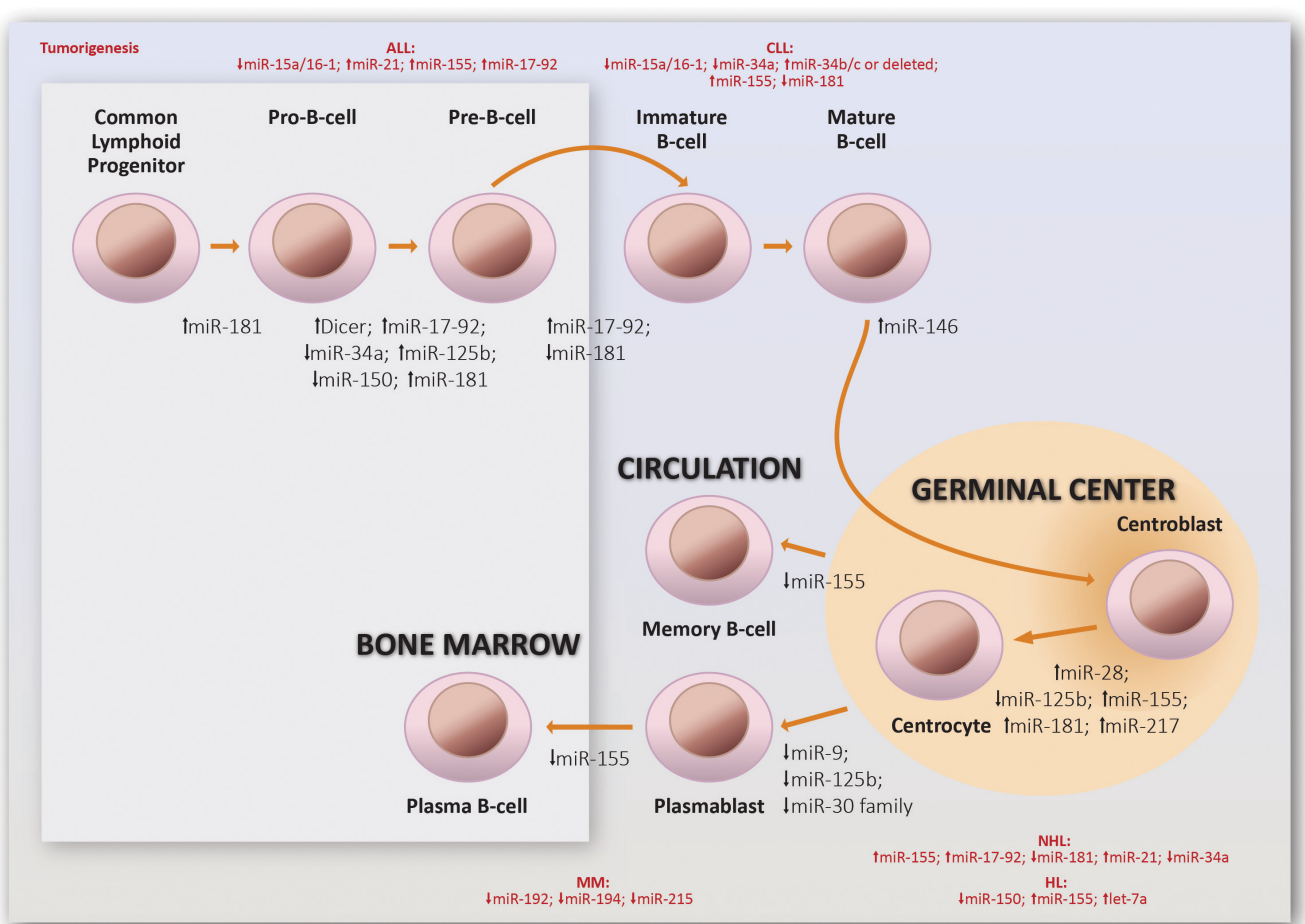
Involvement of miRNAs on normal and malignant human B-cell differentiation The downregulation (↓) or upregulation (↑) of the miRNAs depicted here has been shown to be involved in the reaction to which they are adjacent. ALL: Acute Lymphoblastic Leukemia; CLL: Chronic Lymphocytic Leukemia; HL: Hodgkin Lymphoma; NHL: Non-Hodgkin Lymphoma; MM: Multiple Myeloma

These are clear examples of how distinct B-cell subsets express different patterns of miRNAs, which we believe contributes greatly to their ability to differentiate. Figure 2 illustrates examples of miRNAs important for normal B-cell differentiation and B-cell-derived tumorigenesis [74-76].

### DEVELOPMENT AND PROGRESSION OF CANCER

The development of cancer is a very complex process. During malignant expansion, cells acquire capabilities that allow tumor growth, such as sustained proliferative signaling, the ability to evade growth suppressors and resist cell death, replicative immortality, induced angiogenesis, activated invasion and metastasis, reprogrammed energy metabolism, and evasion from immune destruction. Tumor-promoting inflammation and genome instability and mutation also contribute to malignant proliferation [77, 78].

Due to their ability to control gene expression, deregulated miRNA expression is awarded a central role in oncogenesis. MiRNAs are recognized to function as oncogenes when their upregulation inhibits the expression of tumor suppressor genes, thereby favoring the development and progression of cancer by evading apoptotic and growth suppression mechanisms. Likewise, miRNAs can act as tumor suppressors, preventing the expression of proto-oncogenes and consequently hindering tumorigenesis, e.g., when inducing cell cycle arrest in malignant cells [79]. Their role is not always clear, as is the case of miR-29 in chronic lymphocytic leukemia (CLL) [80]. MiR-29 is downregulated in aggressive CLL causing the upregulation of TCL1, a critical oncogene, suggesting it may act as a tumor suppressor. However, it is involved in the pathogenesis of indolent CLL, where it is expressed at higher levels than in aggressive CLL and normal B-cells, which points to an oncogene function.

The mechanisms causing deregulated expression of miRNAs are not always known. However, Calin et al. [81, 82] showed in 2004 that they are frequently located in fragile sites and in cancer-associated genomic regions. More recent studies have documented epigenetic silencing of miRNAs by DNA methylation and chromatin modifications [83] as well as miRNA targeting of DNA methyltransferases [84, 85], histone deacetylases [86], and proteins involved in histone modifications [84, 85, 87] in multiple cancers. The impact of miRNA regulation of gene expression is illustrated in a more indirect manner when cancer cells truncate the 3′ UTR of target mRNAs by alternative polyadenylation, thus avoiding miRNA regulation [88, 89]. Here we present a number of studies based on either global miRNA expression profiling or functional *in vitro*/*in vivo* models that pinpoint the importance of the deregulated expression of miRNAs in the development and progression of hematological B-cell cancers.

#### miR-15 and miR-16

The first demonstration of the relevance of miRNAs in human cancer recognized the involvement of miR-15 and miR-16 in CLL. With the knowledge that deletions at chromosome 13q14 occur frequently in CLL, lymphomas, and solid cancers, Calin et al. [81] found that miR-15 and miR-16 are localized in a 30 kb genomic region at 13q14 also associated with translocations in CLL [7]. In CLL patients with a deletion of that region, both miRNAs are downregulated compared to normal CD5+ B-cells. The functional evaluation of their activity was provided in a mouse model where the minimal deleted region of 13q14, containing miR-15a/16-1, was conditionally deleted, resulting in CLL phenotype disease [90]. These results point to a tumor suppressor function for miR-15a and miR-16 in CLL (Table 1).

**Table 1:** MiRNA involvement in hematological malignancies

miRNA	Hematopoiesis	Ref	Development and progression of cancer	Suggested mechanism in hematological cancers	Ref	Response to treatment	Ref	Possible protein targets*
miR-15 miR-16	miR-16: regulates differentiation of late erythroid progenitor cells	[159]	Tumor-suppressor in CLL Possibly different roles in early and late-stage MM	Cell cycle arrest Decrease in proliferation Antiangiogenic activity	[7, 81, 91-93]	MiR-15a expression increased in MM cell lines in the presence of bortezomib, decreased when BMSC were added	[92]	Bcl2, cyclin D1, cyclin D2, CDC25A, pRb, AKT3, pAKT, rpS6, pERK, TAB3, VEGF miR-16: RARS
miR-17-92 cluster	Essential for Pro-B and Pre-B-cell survival	[74]	Oncogene in B-cell lymphoma miR-17: Tumor-suppressor in miR-92a-induced erythroleukemia	Decrease in apoptosis (possibly due to elevated myc) Increases cell proliferation Promotes cell survival Promotes the development of stem cell properties or characteristics of early developmental lineages Activation of the PI3K/AKT pathway	[103, 111, 113, 128, 160, 161]	Overexpression increased survival of MCL cell lines after treatment with topotecan, doxorubicin, or etoposide Downregulation suppressed tumor growth in a mouse model of MCL treated with doxorubicin miR-19a and miR-19b were downregulated in MM cell lines in the presence of ITF2357 miR-17: High expression associated with shorter OS in MM patients	[128, 129, 142]	p53, Gata-1, PTEN, E2F1, Bim, PHLPP2 miR-17: Bcl2, STAT-5, Jak2, p21 miR-19a and miR-19b: SOCS1 miR-92: KLF2 [63]
miR-21	---	---	Oncogene in MM and B and T-cell lymphoma	Decrease in apoptosis	[13, 99-104, 162]	Associated with resistance to CHOP in DLBCL	[130]	PTEN, PPCD4, Bim, SHIP1
miR-27a	Promotes erythroid differentiation	[163]	---	---	---	Lower expression in K562 cells resistant to doxorubicin	[134]	MDR1, STAT-5, GATA2 [159]
miR-28	Involved in the GC reaction	[114]	Tumor-suppressor in GC-derived lymphomas	Decrease in proliferation and clonogenicity Increase in apoptosis	[114]	---	---	BAG1, MAD2L1, RAP1B
miR-34a	Delays Pro-B to Pre-B-cell transition Essential for transdifferentiation from Pre-B-cells to macrophages	[164, 165]	---	---	---	In the presence of bortezomib, downregulation of miR-34a increased apoptosis of P493-6 cells miR-34a overexpression in TP53-mutated MM cell lines and mice inhibited tumor growth	[125, 127]	Myc, p53, BCL2, CDK6, NOTCH1, Lef1, Foxp1
miR-122	---	---	---	---	---	Protective action against treatment with bortezomib, MG132, and GSI-1	[139]	cyclin G1
miR-127-3p	---	---	---	---	---	Prognostic role in MCL patients especially in combination with other clinical indicators	[141]	BLIMP1
miR-146b-5p	---	---	Tumor-suppressor in DLBCL	Decrease in proliferation	[143]	Lower expression in CHOP-treated DLBCL patients with poorer OS	[143]	---
miR-150	Blocks Pro-B to Pre-B-cell transition High expression in T-cells Downregulation during erythroid and megakaryocytic differentiation	[64, 66, 159]	Tumor-suppressor in NK/T-cell lymphoma	Increase in apoptosis (especially in the presence of inhibition of miR-21) Decrease in cell proliferation Control of aging and senescence by decreasing telomerase activity	[2]	---	---	DKC1, AKT2, Myb, Survivin, Foxp1, cMYB
miR-155	Controls GC reaction Regulates T helper cell differentiation	[69]	Oncogene in B-cell lymphoma	Decrease in apoptosis	[109, 162]	---	---	PTEN, PPCD4, Bim, SHIP1, FXR1, Ago2, c-Maf, Pu.1, AID, CXCR4, JUN, GATA-3 [159]
miR-181a/b	Early differentiation of B-cells Decrease in proliferation of T-cells Decrease granulocytic and macrophage-like differentiation	[62, 147, 164, 166]	Oncogenes in NHL and AML	NHL: Decrease in apoptosis AML: blockage of myeloid differentiation and infiltration of leukemic cells into the bone marrow and spleen	[144, 145, 147, 148]	Promote apoptosis in fludarabine-exposed CLL cells that express p53 Prognostic potential in CLL showing downregulation during progressive disease	[144, 145]	CXCR4 [63], BCL2, MCL1, TCL1, XIAP, PRKCD miR-181a: Bim, CAMKK1, CTDSPL miR-181b: AID
miR-221 miR-222	Downregulation is essential for expansion of erythroblasts	[115, 159]	Oncogenes in MM	Increase of DNA replication AKT activation	[115, 116, 159]	---	---	p27Kip1, PTEN, p57Kip2, c-kit
miR-223	Involved in the transition from GC cells to post-GC cells Controls granulocytic differentiation Essential for transdifferentiation from Pre-B-cells to macrophages	[36, 63, 165, 167]	---	---	---	---	---	NFI-A, Lef1
miR-320d	---	---	Tumor-suppressor in DLBCL	Decrease in proliferation	[143]	Lower expression in CHOP-treated DLBCL patients with poorer PFS and OS	[143]	---
miR-331-5p	---	---	---	---	---	Lower expression in K562 cells resistant to doxorubicin	[134]	MDR1
miR-615-3p	---	---	---	---	---	Prognostic role in MCL patients especially in combination with other clinical indicators	[141]	---
miR-886-5p	---	---	---	---	---	High expression associated with shorter OS in MM patients	[142]	NR3C1

* Based on references mentioned for Hematopoiesis, Development and progression of cancer, and Response to treatment, unless otherwise stated.

Ago2: Argonaute-2; AID: Activation-induced cytidine deaminase; AKT3: AKT serine/threonine protein kinase-3; BAG1: Bcl2-associated athanogene; DKC1: Dyskerin; FKBP51: FK506-binding protein 51; FXR1: Fragile-X mental retardationrelated protein 1; MAD2L1: MAD2 mitotic arrest deficient-like 1; PDCD4: Programmed cell death 4; PHLPP2: PH domain and Leucine-rich repeat Protein Phosphatase 2; PTEN: Phosphatase and tensin homologue; RAP1B: member of RAS oncogene family; RARS: Arginyl-tRNA synthetase gene; rpS6: ribosomal-protein-S6; SHIP1: Src homology-2 domain-containing inositol 5-phosphatase 1; SOCS1: Suppressor of cytokine signaling 1; TAB3: TGF-beta activated kinase 1/MAP3K7 binding protein 3; VEGF: Vascular endothelial growth factor; XIAP: X-linked inhibitor of apoptosis protein.

Similar results have been found in multiple myeloma (MM). Roccaro et al. [91] identified a miRNA signature for relapsed/refractory MM patients that included downregulation of both miR-15a and miR-16. Additionally, 9 MM patients with chromosome 13 deletion were devoid of expression of these two miRNAs, while 6 MM patients without the deletion presented decreased expression compared to bone marrow from 4 healthy donors. Transfection of MM cell lines with miR-15a and miR-16 simultaneously [91] or with miR-15a alone [92] caused cell cycle arrest and decreased proliferation, supporting a tumor suppressor role. Both studies found an indirect regulation of the vascular endothelial growth factor (VEGF) mature protein, crucial in the pathogenesis and progression of MM, which presented decreased levels after transfection with the miRNAs.

The function of miR-15a in MM has not been completely elucidated since its expression in 52 newly diagnosed patients was found to be higher than in 2 healthy donors [93]. This suggests a different role for miR-15a in primary and advanced MM, illustrating that the function of a given miRNA may depend on cellular context.

#### miR-21

MiR-21 has been reported to be upregulated and to possess oncogenic activity in several cancers, including breast cancer [94], colon adenocarcinoma [3], glioblastoma [95], non-small cell lung cancer [96], esophageal squamous cell carcinoma [97], and melanoma [98]. Overall, miR-21 has been shown to be involved in the development and progression of cancer, with higher expression in more advanced stages of the disease. Although the studies on hematological conditions are limited in number in comparison with solid tumors, miR-21 has also been found to be upregulated in MM, B- and T-cell lymphoma patients and cancer cell lines, including DLBCL [13, 99-103]. Additionally, Medina and colleagues [104] showed that transgenic mice overexpressing miR-21 developed signs of hematological malignancies, which in the majority of the mice resulted in lymphomas of the B-cell lineage. When the overexpression of miR-21 was blocked with doxycycline tumor regression was observed, allowing the authors to suggest oncomiR-21 addiction, with miR-21 impacting not only the initiation of a malignant process but also its development and maintenance, making it an important potential therapeutic target.

Bone marrow stromal cells (BMSC) have been shown to exert a protective effect on MM cells, contributing to their proliferation. Recently, miR-21 was found to be upregulated in MM cells grown with BMSC, with levels even higher than in MM patients. This was observed both in primary patient samples and in the INA-6 cell line compared to normal B-cells [105]. The inhibition of miR-21 with oligonucleotides contributed to an anti-tumor effect *in vitro* in cells with high basal expression and *in vivo* in mouse bearing xenografts of high expression human cells [105]. This suggests that downregulation of miR-21 could potentially be a therapeutic strategy, but not using a “one size fits all” approach.

#### miR-155

GC B-cells express high levels of miR-155, while relatively low expression is observed in pre and post-GC B-cells, indicating a role in the GC reaction [57, 63, 67, 68]. Upregulated miR-155 expression compared to normal counterpart B-cells or tissues was observed for several hematological cancers, including DLBCL of the most aggressive type (ABC-DLBCL) [13, 106, 107], PMBCL, and Hodgkin Lymphoma (HL) [108]. Recently, epigenetically reactivated miR-155 was documented in CLL, where the promoter of miR-155 exhibited a significant decrease in local DNA methylation accompanied by consistent transcriptional upregulation of the mature miR-155 [83]. Functionally, there is evidence of oncogenic properties for miR-155 because its overexpression in transgenic mice consistently caused B-cell lymphoproliferative disease [109].

#### miR-17-92 cluster

The miR-17-92 cluster is located on chromosome 13q31, a region frequently amplified in different tumor types [110]. It encodes 6 miRNAs: miR-17, miR-18a, miR-19a, miR-19b-1, miR-20a, and miR-92-1. It is highly expressed in progenitor B-cells, and expression diminishes as cells mature. The miR-17-92 cluster antagonizes the expression of BIM, a pro-apoptotic protein, thereby favoring the survival of B-cell progenitors [74]. This cluster, as well as individual miRNAs from the cluster, was found to be upregulated in samples from DLBCL, mantle cell lymphoma (MCL), and acute lymphocytic leukemia (ALL) [74, 111, 112]. In a functional *in vivo* murine model of human B-cell lymphoma overexpressing the *c-myc* oncogene, mice with simultaneous overexpression of a miR-17-19b-1 truncated cluster experienced accelerated disease development, which culminated in highly malignant B-cell lymphoma with lower apoptotic capacity and overall survival (OS) [111]. These results suggest that the miR-17-92 cluster has oncogenic properties. However, the function of the individual miRNAs may be dependent on factors such as the lineage of the cells. For example, Li et al. [113] recently reported that miR-17 was able to delay the development of miR-92a-induced erythroleukemia.

#### miR-28

MiR-28 has been shown to be downregulated in GC-derived lymphomas compared to GC B-cells [114]. The authors provided functional evidence of the role of this miRNA in the proliferation of malignant cells, finding reduced proliferation and clonogenicity, increased apoptosis, and G1-arrest when miR-28 was overexpressed. This suggests miR-28 may have a tumor suppressor function. However, these results need further validation.

#### miR-221 and miR-222

Deregulated expression of miR-221 and miR-222 has also been associated with tumor development. Although able to inhibit erythroleukemic cell growth through impairment of erythroid differentiation and proliferation [115], they are reported to possess oncogenic activity. Di Martino and colleagues [116] recently demonstrated their potential as therapeutic targets by showing antitumor activity from miR-221 and miR-222 inhibitors in two MM cell lines expressing moderate or high levels of both miRNAs. The inhibitors were also tested on an MM mouse model where the miR-221 inhibitor, able to downregulate both miRNAs, presented higher activity than the miR-222 inhibitor alone or the two used in combination. This is one of several examples of the possible therapeutic application of the modulation of the expression of miRNAs.

### RESPONSE TO TREATMENT

One of the main reasons for the dismal OS of patients suffering from different types of B-cell-derived cancer is treatment resistance. There are two clinically distinct types of resistance: intrinsic and acquired. The former causes primary refractory disease, reflecting characteristics within the tumor at time of diagnosis that determine the ability of the tumor cells to thwart the effectiveness of a given drug. In contrast, acquired resistance occurs in initially responsive patients, and it is caused by a selection process where chemoresistant clones overgrow the sensitive ones [117-119]. Early detection of resistance is of great importance. Therefore, there is a strong need for biomarkers that ensure a more accurate classification of B-cell-derived malignancies or that are able to guide selection of alternative treatments. Defining miRNA involvement in resistance will be useful to guide treatment selection and help monitor treatment administration. It could provide information on the mechanisms activated by the drug and make miRNAs themselves biomarkers of both intrinsic and acquired chemoresistance, thereby improving primary treatment choice and circumventing intrinsic resistance.

MiRNAs can act as prognostic and predictive biomarkers. The prognostic or predictive role can be explained by the anticipation of how a patient is expected to respond to a combined intervention or to one drug in particular, respectively, based on the presence or absence of a miRNA. Understanding the involvement and the possible use of miRNAs in response to treatment is invaluable to tailor the therapeutic approach to each individual patient. In the following two sections we will present the potential of miRNAs in guiding treatment strategy. We first present studies that show how the *in vitro* or *in vivo* manipulation of the expression of specific miRNAs can affect the response to a given drug, and consequently unravel their biological roles in treatment response by pinpointing the potential of miRNAs as predictive biomarkers. In the second section we will focus on miRNAs that have demonstrated a correlation between expression levels and outcome of treatment, illustrating the role of miRNAs as prognostic biomarkers.

#### MiRNAs as predictive biomarkers of treatment response

The knowledge of how a patient is expected to respond to a specific drug or regimen is very helpful in regard to deciding the best possible treatment. It can prevent the occurrence of toxicity or sub-therapeutic dosages, or indicate that another therapeutic regimen may be more suitable for the patient in question. This information is provided by predictive biomarkers. Understanding the biological function of miRNAs may suggest that a specific drug is effective or not. However, the study of predictive biomarkers, which provide limited biological insight, is extremely important as it can more strongly support the development of individualized treatment strategies.

#### miR-15 and miR-16

MiR-15 and miR-16 have been linked to sensitivity to chemotherapeutic agents. Hao et al. [92] showed that MM U266 and H929 cell lines were sensitive to bortezomib and the expression of miR-15a was increased in the presence of the drug. When the bortezomib-treated cells were allowed to interact with BMSC the tumor suppressor effect was decreased along with miR-15a levels. The expression of the anti-apoptotic protein Bcl-2, a target of miR-15a, was upregulated in these cells contributing to the protection of MM cells against bortezomib-induced apoptosis and promoting their survival. As mentioned earlier, transfection with miR-15a and miR-16 promotes cell cycle arrest and inhibits proliferation of MM cell lines [91, 92]. Therefore, induction of these miRNAs may be one of the mechanisms of action of bortezomib. These studies suggest that overexpression of miR-15a and miR-16 can help overcome resistance towards bortezomib in MM.

#### miR-34a

The role of miR-34a in cancer has not been completely clarified. Although there are publications suggesting a tumor-favoring activity, the majority of studies point to a tumor suppressor function. Hence, activity most likely depends on the cellular and genetic microenvironment [120, 121]. The miR-34a gene is located on chromosome 1p36.22, a region frequently altered in cancer [82]. Its promoter can become hypermethylated, causing reduced levels of miR-34a [122]. Downregulated miR-34a expression is observed in gastric marginal zone B-cell lymphoma of MALT type that transforms into gastric DLBCL [123]. Reestablishing miR-34a expression in a lymphoma xenograft model reduced tumor growth by 76%, suggesting a true therapeutic utility of this miRNA [124]. Accordingly, miR-34a overexpression in *TP53*-mutated MM cell lines and in mice bearing xenografts of these cells showed tumor growth inhibition and even regression, supporting a tumor suppressor role when *TP53* is mutated [125]. However, the genetic and deregulated expression background of the B-cell tumors may dramatically affect the function of miR-34a. On one hand, miR-34a is able to mitigate resistance to taxol and platinum-based agents in solid tumors [126]. On the other hand, Sotillo et al. [127] showed that transfection of miR-34a into a B-cell line genetically similar to Burkitt's Lymphoma, overexpressing Myc, downregulated Myc and p53 protein levels, which led to decreased apoptosis. In the presence of bortezomib, upregulation of miR-34a inhibited p53-dependent drug-induced apoptosis. MiR-34a downregulation increased the apoptotic activity, illustrating that in tumors with deregulated Myc expression miR-34a confers drug resistance and its downregulation could be a strategy to increase response to bortezomib. Thus, the initial mutational status and subsequent treatment-induced clonal selection of malignant cells affect the intrinsic and acquired drug resistance, respectively, and also the miRNAs involved in the drug response.

#### miR-17-92 cluster

Treatment outcome of MCL patients may also be influenced by miRNA expression. MiRNAs of the cluster miR-17-92 were shown to be upregulated in these patients, with 2 to 3-fold greater levels than in normal naïve B-cells. High levels of *C13orf25*, the gene that encodes the cluster, were correlated with poorer OS [128]. Overexpression of the cluster by stable transduction in MCL Z138c and Granta-519 cell lines increased cell survival after exposure to topotecan, doxorubicin, or etoposide, while knockdown of miR-17-92 was shown to significantly suppress tumor growth in a mouse model of MCL treated with doxorubicin. These miRNAs also impacted the response to treatment of other cells. In the presence of the histone deacetylase ITF2357, a drug that induces apoptosis in MM cells, miR-19a and miR-19b were 25-57% downregulated in KMS18 and KMS12 cells [129]. This was reported as a possible mechanism of action of the drug. Downregulation of miR-17-92 might therefore be beneficial to reduce treatment resistance in MCL.

#### miR-21

MiR-21 was recently suggested to play a part in resistance to the CHOP (cyclophosphamide, doxorubicin, vincristine, and prednisone) regimen in DLBCL, as its knockdown in CRL2631 cells increased the sensitivity to treatment via phosphatase and tensin homolog (PTEN) upregulation [130]. This is in agreement with observations in MM, where miR-21 inhibition in combination with dexamethasone, doxorubicin, or bortezomib inhibited myeloma cell survival more effectively than any treatment alone [131]. Together with the findings of upregulation of miR-21 in DLBCL, MM, and several other cancers, these results support its oncogenic role and potential as a therapeutic target. Simultaneously, diminished expression of miR-21 might be predictive of tumors likely to be responsive to CHOP, dexamethasone, doxorubicin, or bortezomib.

#### miR-155

Epstein-Barr virus (EBV)-associated DLBCL is a rare disease with poor survival due to resistance to chemotherapy [132]. EBV expressing latent membrane protein-1 (LMP1) activates the Akt pathway by phosphorylation and upregulates the antiapoptotic MCL1 through miR-155 expression, which contributes to the survival of the rituximab-treated cells. This shows the involvement of miR-155 in resistance to rituximab. Knockdown of miR-155 expression is able to reduce Akt phosphorylation, resulting in the significantly increased death of EBV-positive cells treated with rituximab [133]. These results indicate that inhibition of miR-155 could be a valuable approach for the treatment of EBV-associated B-cell lymphomas.

#### miR-331-5p and miR-27a

Three myelogenous leukemia cell lines derived from K562 cells with gradually increasing resistance to doxorubicin showed an inverse correlation between the expression of two miRNAs, miR-331-5p and miR-27a, and the degree of resistance to the drug [134]. When transfected with these miRNAs, the doxorubicin-resistant cells exhibited a higher sensitivity to the drug and decreased expression of P-glycoprotein (P-gp). This could be due to the targeting of the multiple drug resistance protein 1 (*MDR1*) gene, which encodes for P-gp and whose increase is identified as a possible mechanism of resistance. In fact, expression of *MDR1* can be increased in B-cell lymphoma Daudi cells by treatment with doxorubicin, which is an example of the development of acquired resistance [135]. Additionally, Raji cells previously co-cultured with follicular dendritic cells presented higher *MDR1* expression and a lower apoptotic rate after exposure to doxorubicin compared to Raji cells cultured alone [136]. Feng et al. [134] also found that relapsing leukemia patients had lower expression of miR-27a and miR-331-5p than patients at diagnosis or in complete remission, supporting the tumor suppressor role of these miRNAs and their possible application in surpassing resistance to doxorubicin.

#### Predictive miRNAs in therapeutic intervention

The predictive potential of miRNAs is directly linked to their ability to be used in the treatment of malignancies. MiRNAs shown to contribute to resistance to a particular drug or regimen are potential targets for treatment, thus, obvious candidates for drug discovery, while miRNAs that directly or indirectly increase the response to treatment have therapeutic potential on their own. The latter are plausible candidates for studies of drug formulation. Therefore, the study of predictive biomarkers provides information on how to guide the treatment of patients with the options currently available and also on possible future therapeutic strategies.

#### miR-122

MiR-122 is so far the only miRNA that has been directly targeted in a clinical trial. It is involved in the cholesterol and lipid metabolism and also in hepatitis C virus replication [137]. Antagonization of miR-122 is being evaluated for the treatment of hepatitis C virus infection in human clinical trials [138]. Initially thought to be liver-specific, miR-122 was later shown to be expressed in cutaneous T-cell lymphoma (CTCL) cell lines [139]. Its overexpression in these cells exerted a protective action against chemotherapy-induced apoptosis, namely against bortezomib, MG132 (proteasome inhibitors), and GSI-1 (a γ-secretase inhibitor). The exact mechanism is unknown; however, Akt and p53 seem to be involved. The results suggest that inhibiting miR-122 may be a strategy to improve treatment outcome in CTCL patients [139].

#### MiRNAs as prognostic biomarkers of treatment response

The potential of miRNAs as prognostic biomarkers under homogeneous treatment, even when their biological roles are not validated, should not be disregarded as this has a direct utility in clinical practice. The anticipation of how a disease will develop is of tremendous importance as it can make a difference in the choice of the treatment course, chemotherapy, or palliative care. It is, understandably, a question patients would like to have answered not only at time of diagnosis but throughout the progression and treatment of the disease. Research for prognostic tools is essential for diseases like cancer that have such a dramatic impact on quality of life and survival. To the best of our knowledge, no single miRNA or panel of miRNAs is at present implemented as validated biomarkers of prognosis in a routine clinical setting, not even when the translation potential is suggested as in the studies mentioned below.

The study of prognostic biomarkers, which offer less biological insight, may initially seem less relevant than studies of predictive biomarkers. However, by showing a correlation with clinical outcome, prognostic biomarkers demonstrate potential utility in a clinical setting and their use is reliable even without fully understanding the mechanism by which they function.

#### miR-222

After identifying a miRNA signature in cell lines able to distinguish ABC from GCB DLBCL, Alencar and colleagues [10, 11] set out to find which miRNAs of the signature correlated with survival in DLBCL patients. In a cohort of 176 patients treated with R-CHOP (rituximab added to the CHOP regimen), they found that increased miR-18a was associated with shorter OS, high miR-222 correlated with shorter progression-free survival (PFS), and increased miR-181a was positively associated with PFS. Shortly afterwards, Montes-Moreno et al. [12] published their findings on a prognostic miRNA signature found in a 36-patient discovery cohort and tested in 240 patients. It comprised 9 miRNAs and was able to predict poor clinical outcome in patients treated with the same regimen as in the Alencar et al. study. MiR-222 was present in both signatures, reinforcing its potential as a prognostic biomarker.

#### miR-34a

MiR-34a has also been correlated with clinical outcome. It was found to be downregulated in DLBCL samples. Although the study did not find an association between miR-34a and OS, the combination of low miR-34a and high FOXP1, BCL2, and p53, validated targets of miR-34a [126], was associated with poorer OS in a multivariate survival analysis [140]. The lack of association with miR-34a alone could be due to the different therapeutic approaches used among patients. This study suggests that several factors are involved in the activity of miR-34a, reinforcing the relevance of the characteristics of the tumor and its surroundings on the activity of this miRNA and supporting a classification of diseases based on its COO.

#### miR-127-3p, miR-615-3p, miR-17, and miR-886-5p

Using a training set of NHL, Goswami and colleagues [141] identified an 11-miRNA signature that distinguished indolent from aggressive forms of lymphomas, highlighting the diagnostic potential of miRNAs. Two of these, miR-127-3p and miR-615-3p, seemed able to provide information on the prognosis of MCL patients, especially if used in combination with the current clinical indicators Ki-67, a marker of cell proliferation, used with a redefined cut-off, or the MCL International Prognostic Index. Wu et al. [142] identified a classifier comprising 2 miRNAs, miR-17 and miR-886-5p, that distinguished three subgroups of MM patients with different OS, improving the classification provided by the International Staging System and Fluorescence In-Situ Hybridization-based abnormalities. Therefore, the combination of miRNA expression with already established prognostic biomarkers is promising for clinical use.

#### miR-146b-5p and miR-320d

A recent report has shown that miR-146b-5p and miR-320d have potential to discriminate between CHOP-treated DLBCL patients with different clinical outcomes [143]. Low expression of both miRNAs compared to the median was found in patients with poorer PFS and/or OS. In addition, miR-320d was more highly expressed in GCB-DLBCL patients.

#### miR-181a/b

MiR-181a and miR-181b also hold promise as prognostic biomarkers. MiR-181b has been shown downregulated in CLL patients and its expression further decreases with progression of the disease [144, 145]. Both miRNAs target anti-apoptotic proteins in these cells and induce apoptosis in primary cell cultures where p53 is normally expressed [145]. In a meta-analysis performed recently, downregulated expression of miR-181a/b was correlated with poor OS in hematological malignancies [146]. However, this could be another situation where the microenvironment deeply affects the activity of miRNAs, as inhibition of expression of the miR-181 family improved the condition of acute myeloid leukemia (AML) mice models and high miR-181a was suggested to behave as an oncogene also in NHL cell lines and primary lymphoma cells [147, 148].

#### Dicer

The impact of deregulated expression of Dicer, essential for the biogenesis of miRNAs, has also been examined. Zhou et al. [93] found that its knockdown in one myeloma cell line led to cell cycle arrest and increased apoptotic events, and Adams and colleagues [149] showed that B-cell lymphomas require Dicer for survival. However, Sarasquete et al. [150] showed an association between its upregulation and longer PFS in symptomatic MM patients. Hence, the effect of changes in the expression of this endoribonuclease on prognosis is not yet clear. This is supported by a recently published review that assessed the prognostic significance of its expression in different types of tumor, showing that different results have been found not only between distinct diseases but also among studies focusing on the same disease [151]. It is necessary to conduct large studies to make statements on how Dicer, and other catalyzers of miRNA biogenesis, may impact or predict the development of diseases.

### PERSPECTIVES

MiRNAs are molecules of the utmost biological importance, making them interesting as diagnostic, prognostic, and predictive biomarkers. In this review, we have presented examples of how they could be useful in clinical practice. Certain miRNAs, specifically miR-15, miR-21, and the miR-17-92 cluster, have been associated with the development or progression of hematological malignancies and response to treatment. The exciting potential of these miRNAs suggests that more clinical studies should focus on them so that their measurement can have clinical utility in the years to come.

However, we have also shown that the results are sometimes contradictory. This might be explained by several factors, including the degree of cellular differentiation or the progression of disease at the moment of sample collection, the sampling material and its quality, the number of samples analyzed, the cellular context, scientific approaches, and statistical analyses. Another factor that may impact the different results, or the lack of associations in many studies, is the fact that miRNAs are usually expressed at low levels. A small fold change may be sufficient to impact the expression of their targets and the networks they interact with but may not be easily detected.

Instead of tumor biopsies, serum and plasma are sometimes used for miRNA analysis. One example is the high expression of 3 miRNAs (miR-494, miR-1973, and miR-21) observed in plasma from classical HL patients before treatment, decreasing to levels similar to healthy individuals at remission [152]. Sevcikova et al. [153] found that miR-29a, normalized against miR-16, was differentially expressed between serum from 91 MM patients and 30 healthy donors, although no association was identified with disease stage. A five-miRNA serum signature with predictive potential towards R-CHOP treatment in DLBCL patients has also been recently reported [154]. There is not always a clear correlation between miRNA expression in these fluids and tumor tissue [152], which according to some authors may be because a tumor would have to develop for several years before the expression of circulating miRNAs could be used as a tumor biomarker [155]. There can also be differences in measurements between serum and plasma, which can be due to the method of extraction of miRNAs. The presence of cellular components and hemolysis affects miRNAs differently in blood fluids [156], highlighting the importance of improvements in the technique. However, the stable expression of miRNAs in serum and plasma and their easy access in these biofluids make their potential as biomarkers evident. This is also supported by the recent study by Chen W et al. [157], who found a linear correlation between miR-21 expression in serum and tumor tissue in 30 DLBCL patients. Additionally, based on serum from 62 patients, they found higher miR-21 levels in patients with a better prognosis.

MiRNA isolation and measurement with PCR in bone marrow smears has been recently reported [158]. The authors found differentially expressed miRNAs between 3 normal donors and 5 FL patients. This type of sample presents the advantage of being easily stored and transported at room temperature. These results suggest that bone marrow smears could be another option for screening miRNA expression.

As shown above, miRNAs are involved in many cellular processes. They are essential in the differentiation, maturation, and metabolism of cells, capable of controlling gene expression and the expression of other non-coding RNAs. This pervasive activity leads us to hypothesize that they also play a role in defining the COO of B-cell malignancies and drug-specific resistance. We hypothesize that the characterization of B-cell subsets will help classify B-cell malignancies and predict their response to treatment. To test this, we are characterizing primary B-cell subsets purified from various lymphatic tissues. Additionally, we have performed *in vitro* drug screens in DLBCL and MM cell lines and scrutinized their mRNA and miRNA expression to identify drug response profiles. Tracing the COO of tumors will allow a better insight into the cancer biology and a more thorough characterization of the tumor of each patient. The combination of this knowledge with associations between expression of specific miRNAs and drug response will enable the prediction of the most beneficial treatment for each patient.

Although the role of specific miRNAs in the development of cancer, response to treatment, and development of resistance still needs further clarification, their potential in therapeutic intervention and as prognostic and predictive biomarkers is undeniable. It is our hope that future studies on miRNAs will provide support for the assignment of individual patients to specific drug response profiles and the consequent development of personalized treatment strategies.
